# A Framework for Improving Policy Priorities in Managing COVID-19 Challenges in Developing Countries

**DOI:** 10.3389/fpubh.2020.589681

**Published:** 2020-10-14

**Authors:** Golam Rasul

**Affiliations:** International Centre for Integrated Mountain Development, Lalitpur, Kathmandu, Nepal

**Keywords:** COVID-19 pandemic, health crisis, developing countries, policy prioritization, policy coordination, sustainability

## Abstract

The COVID-19 pandemic has brought unprecedented challenges to societies and threatened humanity and global resilience. All countries are challenged, but low-income and developing countries are facing a more challenging situation than others due to their limited health infrastructure, limited financial and human resources, and limited capacity of governments to respond. Further, the interconnected nature of the COVID-19 pandemic crisis demands an integrated approach and coordinated action, which complicates decision making even more. Identifying the best set of policies and instruments to address COVID-19 challenges, and aligning them with broader social goals will be critically important for sustainable recovery from the pandemic. The key practical challenge facing the policy makers of developing countries is how to prioritize policies to achieve the interconnected goals of managing the health crisis, recovering the economy, and achieving environmental sustainability. We present a framework for identifying and prioritizing policy actions to address the COVID-19 challenges and ensure sustainable recovery. The framework outlines principles and criteria and provides insights into developing shared policy goals, identifying smart strategies, assessing policy compatibility, aligning policy instruments, and factoring sustainability into short and long-term policy decisions. This framework can assist policy makers in linking short and long-term goals, mapping the interactions of different policy options, and assessing anticipated consequences and cross-sectoral implications. This will enable policy makers to prioritize policy choices and allocate limited resources in such a way that they are directed toward actions that generate synergy and co-benefits, have multiplier effects, and achieve interconnected solutions for health, the economy and environment.

## Introduction

From a health crisis, the COVID-19 pandemic has become a “systemic global risk” (1). The virus is highly contagious and spreading fast. It does not recognize borders, spares no one, and permeates all aspects of our lives and well-being. It has affected healthcare and economic and social norms and values, has taken many lives, and has threatened the livelihoods of billions of people. It has brought unprecedented challenges to societies and threatened humanity and global resilience. All countries are challenged, but low-income and developing countries are facing a more challenging situation than others due to their limited health care facilities, low human capital, high poverty, and limited capacity of governments to respond effectively to such a pandemic (2).

Many developing countries have poor health care systems; more than 70% are among those “least prepared” for a pandemic with a global health security index score of <40 out of a 100 (3). In South Asia, Afghanistan has only 2.8 physicians per 10,000 people, Bhutan 3.8, Bangladesh 5.3, and Nepal 6.5, a tenth of the number in more advanced countries. Even India, which has one of the strongest health systems in the region, has only 7.8 physicians per 10,000 people (3). The situation is even worse in many African countries. And there are similar problems with health facilities and physical and human resources. For example, Malawi has only 25 critical care beds for 19 million people and many counties in Kenya have no functioning ventilators (4).

In most developing countries, the challenges of coping with and slowing the pandemic are compounded by adverse social conditions. Not only are the health care systems weak, many people have no health insurance or social security (1). Two of three workers are in the informal economy with no employment contract or social security and only limited or no savings to meet healthcare costs or even basic human needs during the lockdown period without borrowing or selling productive assets (5). In many countries, people lack access to basic services such as clean water, sanitation, and hygiene facilities. For example, close to 42% of households in Afghanistan are compelled to use unsafe drinking water and more than 50% do not have access to water and soap for washing hands (1). Furthermore, high population densities, poor working conditions, and inadequate living space make social distancing very difficult. About a billion people, most of them in developing countries live in urban slums and informal settlements (6–8). Many of these are home to huge numbers of people, for example the Orangi area in Karachi, Pakistan (2.5 million), Dharavi in Mumbai, India (1 million), Neza in Mexico (1.2 million), Kibera in Kenya (0.7 million), Khayelitsha in Cape Town, South Africa (0.4 million), and the Rohingya camps in Cox's Bazaar, Bangladesh (about 1 million). These overcrowded living spaces and limited—often shared—water and sanitation facilities have made physical distancing and self-isolation difficult and increased the risk of exposure and vulnerabilities.

With weak health infrastructure and limited financial and human resources, strategic thinking and planning and setting priorities for policies and activities will be critically important for developing countries to manage COVID-19 challenges (1, 2, 9). Identification of policy priorities and selection of appropriate policy instruments is one of the more powerful means for policy success (10). However, decision making and prioritization has always been a challenge, and the uncertain and volatile nature of the COVID-19 crisis has further complicated the issue (1, 2, 11–13). Further, the interconnected nature of the crisis demands an integrated approach and coordinated action, which complicates decision making even more (14, 15). The key practical challenge facing the policy makers of developing countries is how to prioritize policies to achieve interconnected goals of achieving health and well-being (9, 16). A clear framework will be needed to ensure effective policy development and prioritization in planning and management of their response. In the following, we suggest an approach and framework that can enable developing countries to develop an effective prioritization process.

## A Framework for Prioritizing Policies and Improving Policy Coherence

**Figure 1:**
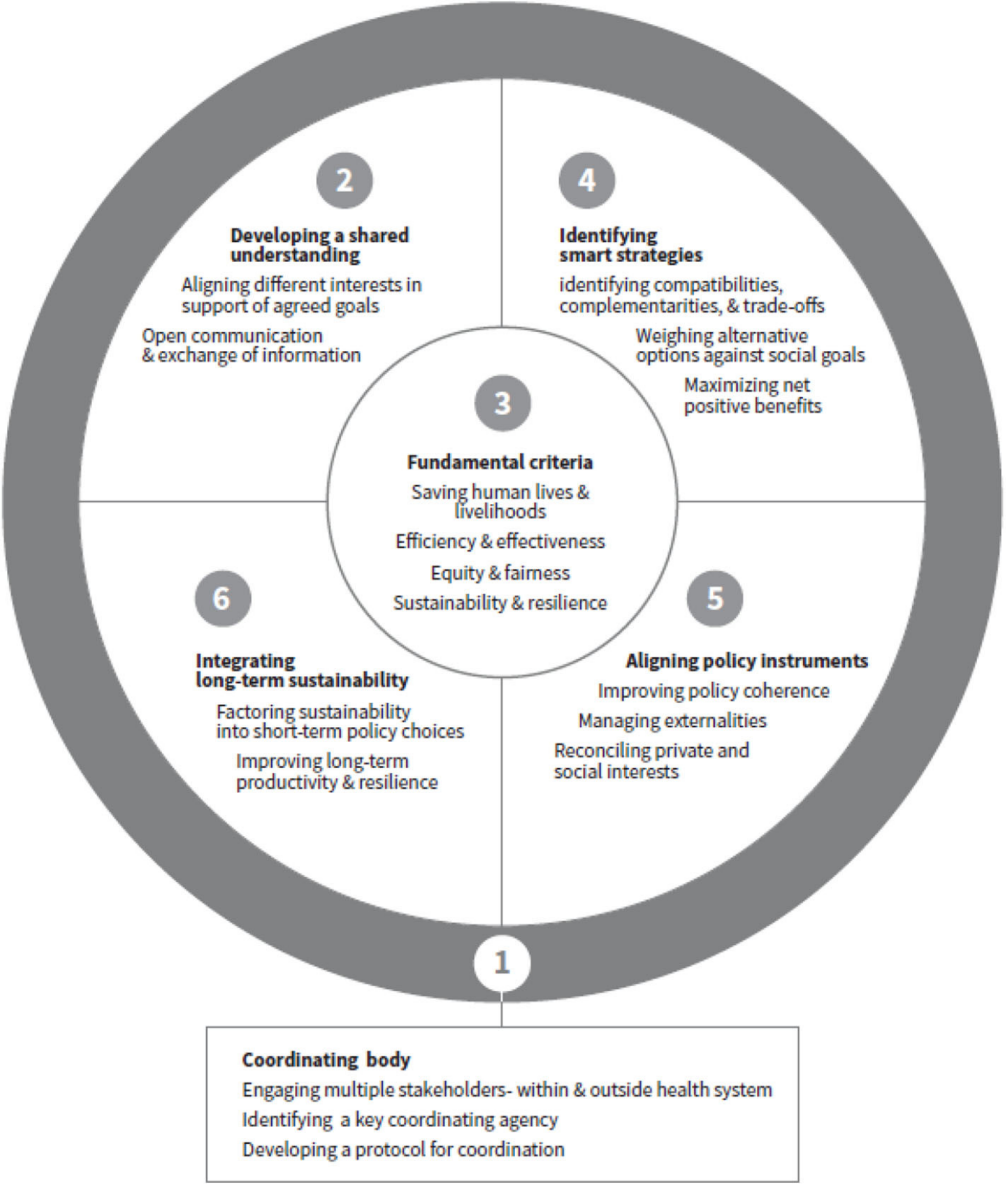
A framework for prioritizing policy choices.

The starting point in setting priorities should lie in engaging and consulting with key stakeholders in order to create a common vision for health, well-being, economic security, and environmental safety, with buy-in from stakeholders and commitment on broad social goals, which is key for implementation effectiveness. The suggested steps are outlined below and the key elements and supporting structures are presented in Figure 1. The framework is developed drawing concepts from public administration, public health, economics, and sustainable development and intended to assist policy makers to weigh policy options and prioritize policy choices within health and outside health sectors for governing complex interconnected issues. There are two major prerequisites for using the framework. The first is to establish a cross-sectoral coordination body, and the second to establish the criteria for assessing and prioritizing policy actions. The principles and criteria for setting priorities identified below are the fundamental basis for weighing different policy choices. These four criteria are at the center of the framework and related to all four steps. The individual elements are described in more detail in the following sections.

### Establishing a Multi-Sectoral Coordination Body and Mechanism

Leadership is critically important for effectively dealing health crisis like pandemic as well as engaging and coordination diverse actions and stakeholder in achieving broader social goals (17). A multi-sectoral coordination body can provide an effective pathway for engaging multiple stakeholders, and the basis for a mechanism for coordinating and steering the decision-making process, and overseeing the implementation and recovery packages to maximize impact (18). Many agencies important for health crisis management work within the health system, ranging from health education to prevention, and protection to treatment. Outside the health system, food and nutrition, water and sanitation, housing, and elements of the physical environment such as air quality and climate are important for maintaining, improving, and protecting health (11, 12, 19). A coordination body could establish a mechanism and develop a protocol for coordinating both the administrative (functional) and the policy (strategic) activities of the different organizations. While the focus of functional coordination could be on building consensus and ensuring smooth cooperation within and between the key organizations involved in the planning process, policy coordination should focus on improving policy coherence and developing consistent policies to improve synergies. Effective administrative coordination is a precondition for successful policy coordination. While detailed consultation is not possible during a time of urgency like the COVID-19 pandemic, this body should be able to engage and consult with key organizations and stakeholders in order to facilitate exchange of ideas, dialogue, and discussion, and provide strategic directions and clear guidelines in setting priorities, allocating resources, and implementation. The key stakeholders could be key government agencies such as health (including public health experts), finance, security, water, food, and trade and commerce, as well as experts and development partners, think tanks, and both government and non-government actors. Effective stakeholder engagement relies on appropriate institutional frameworks as well as effective use of both formal and informal coordination mechanisms to facilitate mutual communication and collaboration among stakeholders and oversee the priority setting process (18, 20, 21). The overall coordination can remain under the cabinet or national planning commission (who have the authority to mobilize sectoral ministries and agencies), or an inter-ministerial committee. However, this can vary considerably from country to country depending on the cultural context and administrative and operational capacity (13). It could be a single structure or unit such as the Ministry of Health or the Office of the Prime Minister or President or Planning Commission. However, it is critically important to identify a key coordinating agency within the current institutional framework, which has the authority to convene and coordinate multi-sectoral actions and guide integrated planning. The coordination body should have broad representation and may include both political leaders and experts in the relevant fields, and should have adequate convening and decision-making power and authority, as well as the capability to deal with strategic issues including policy coordination. This requires a continuous process of analyzing, balancing, and prioritizing the objectives of different policy goals. It may also require enhancing institutional capacity, including the operational and coordination skills of coordination agencies, and improving processes that facilitate engagement with stakeholders beyond governments, such as private health service providers and other non-state actors (20, 21). In striving to establish multi-sectoral coordination, it is also important to distribute responsibility and establish mechanisms for regular interaction among key stakeholders in order to build inter-organizational trust and promote communication and share knowledge and information among key institutional agencies.

### Developing a Shared Understanding and Objectives

One of the fundamental steps (Figure 1) in the planning and prioritization process is breaking sectoral silos and aligning interest of different agencies to develop a shared understanding on policy goals and strategic objectives and short and long-term needs of a country (18). This provides the basis for achieving a consensus on multi-pronged strategies to achieve short-, medium-, and long-term goals. To avoid conflicts, decision making on strategic policy choices and programs needs to be mindful of the broader social goals and their potential conflicts. The short-term focus will be on managing the immediate health crisis, ensuring food and nutrition, and short-term job creation to help the economy survive; the medium-term on boosting economic activities to achieve financial and economic recovery; and the long-term on transforming or bouncing the economy forward by promoting long-term sustainable growth, reducing inequalities, building social coherence and resilience, and conserving resources and protecting the planet. The different goals are closely linked and interdependent as health, economic, social, and environmental systems are interconnected (22). The shared policy goals provide a basis for developing practical criteria and guidelines for prioritization.

#### Building Consensus Among Agencies

*Building consensus among agencies* within and outside of the health system is a daunting task. It involves engagement with the relevant government agencies and other key stakeholders and reaching societal agreement on common priorities that reflect the views of key stakeholders including epidemiologists and economists (15). This not only provides the fundamental basis for prioritization but also creates buy-in, commitment, and accountability from stakeholders on the broad social goals. Although different government agencies have different interests and priorities, facilitating discussion and consultation, mediating conflicts, building trust, and providing a platform to clarify expectations can enhance mutual understanding and align interests (21). One way to align multiple perspectives and build shared understanding through the process of engagement known as “principled engagement” that fosters reasoned argument (weigh different options and priorities objectively against broad social goals) and deliberation focused on defining problems and finding agreements together (23). It supports shared representation and open interactions of different sectoral actions, and to integrate the concerns and goals of different sectors and agencies. It allows open discussion, surface multiple perspectives, and enables “shared motivation” that build trust, foster mutual recognition of interdependence and shared ownership, and create a sense of internal legitimacy (23). The principled engagement and shared motivation support each other and create an enabling environment for integrated planning, jointly identifying and defining objectives as well as a collaborative, raising awareness about the complementarities and externalities, and using a coordinated approach to consultation with open communication and exchange of information, will help align multiple perspectives reduce disagreements, increase understanding, and clarify organizational responsibilities (14, 18). Besides the four fundamental criteria presented above, aligning the different interests in support of agreed goals and building consensus through open communication and effective collaboration is critically important for policy prioritization in managing COVID-19 challenges and recovering the economy sustainably. In striving to build a consensus on policy goals and maintaining shared understanding, the interests, needs, and positions of different stakeholders need to be understood and assessed based on the fundamental criteria outlined above.

### Agreeing on the Principles and Criteria for Setting Priorities

Priority setting is a complex process involving making decisions on the allocation of resources to improve policy goals. The interests, motivations, and preferences of the diverse array of stakeholders will differ, thus prioritization needs to be based on explicitly chosen and agreed criteria.

#### Dimension of Priorities

Prioritization is a multidimensional concept and can be seen from different perspectives, all of which need to be understood and taken into account. From a moral and ethical perspective, managing existing, and emerging threats to human lives is a societal obligation and the primary responsibility of states (24, 25). Thus, the highest priority should be given to policy choices that save human lives by reducing health risks, improving health care, reducing communicable diseases, and ensuring provision of basic health services, together with those aimed at meeting basic human needs such as access to food, water, and shelter. From a utilitarian perspective, policy choices should be guided by the utility generated and cost-effectiveness, since resources are finite (26, 27). Thus, the highest priority should be given to the policy choices that are most cost-effective and generate the maximum net social benefits. From an egalitarian perspective, equity, and fairness are equally important in policy choices (28, 29). Cost-effectiveness is important but should not be the sole criterion; an equally high priority is given to protecting those who are most at risk and serving the most deprived even if this is less cost-effective. From a resilience perspective, present actions should prepare for transition to a more resilient and better society (12, 30). Thus, a high priority should be given to policy options that enhance long-term social, economic, and environmental benefits that lay the basis for long term resilience and build the capacity to deal with future challenges.

The basic principles and criteria should be agreed by the key stakeholders and effectively communicated across all stakeholders. Using these broad perspectives, four practical criteria can be identified as the fundamental basis for assessing and prioritizing policy choices and thus allocating resources (Figure 1):

- Saving human lives and livelihoods
- Efficiency and effectiveness
- Equity and fairness
- Sustainability and resilience

These four principles and criteria may vary from country to country and different countries may attach a different weight to the different criteria based on the socio-economic conditions, existing health facilities, financial capacity, and environmental conditions of the country and the specific social, economic, and environmental concerns.

### Identifying Smart Strategies That Bring Synergistic Effects

Once agreement has been reached on policies and prioritization criteria, the next step is to develop strategies for integrated and coordinated implementation of the different policy measures (Figure 1). It is important to explore complementarities and identify potential co-benefits for the different policy options that bring synergistic effects by achieving multiple objectives at the same time with benefits for both health and economic recovery.

In addition to the fundamental criteria for assessing priorities, developing countries generally prioritize providing jobs and income for the poor and vulnerable (31). Thus, a typical smart strategy could comprise investing in labor-intensive sectors that immediately generate employment while also generating multiplier effects for the economy by increasing growth potential and supporting economic recovery (31, 32). For example, investment in public works, infrastructure, small business, and micro and small enterprises can quickly offer jobs and income while stimulating local economies through using local resources and increasing the demand for manufactured goods (31, 32). The outcome of such strategies, however, will depend on the local conditions and the way in which the programs are designed and implemented.

In addressing the challenges brought by the COVID-19 pandemic, it is important to choose policy options that support, and don't reduce, achieving other strategic objectives (33). Identifying such options involves analyzing the interactions among different strategies, assessing the magnitude and nature of benefits, and identifying compatibilities, complementarities, and trade-offs. For example, access to clean water, sanitation, and hygiene is critically important for addressing the challenge for COVID-19 health risks, so investment in water infrastructure can generate employment and provide health benefits. In contrast, economic activities that pollute water and air undermine efforts toward achieving human health. Similarly, investment in education is key for reducing economic vulnerability and developing resilient systems. Education and health improve human capital, while human capital shapes productivity (34). The World Development Report estimates that those developing countries with low human capital today, will have a future workforce that is only one-third to one-half as productive as a workforce in full health and having a good education (34). Policy support to empower socially disadvantaged communities to exercise control over the social and economic factors that determine their health can improve long-term health benefits (11). Policy choices that increase access to health and education, develop new skills, improve productivity, improve air, water, and soil quality, conserve natural resources, build adaptive capacity, and reduce inequalities all help to improve socio-economic resilience, whereas policies aimed simply at achieving short-term gain can result in unsustainable practices that reduce long-term adaptive capacity and affect planetary health (32, 35). Policy options that bring synergistic effects with other strategic objectives should get priority. For example, a policy choice to create jobs in rural areas through growing nutritious crops can create jobs for the unemployed in agriculture and also support the objective of achieving good health through nutrition (36). Policy options that constrain achieving other goals or undermine long-term resilience should be avoided or minimized as far as possible.

#### Improving Policy Coherence

While in certain areas policy cannot be compromised, there are many areas where improving policy coherence and coordination can reduce trade-offs and improve synergies and thus increase the net positive gain to society (19, 32). It is therefore critical to assess the magnitude of trade-offs and find ways and means to minimize them and improve the net positive outcome on the broad social goals outlined in the basic principles. For example, relaxing lockdown may increase the risk of spreading the virus, but properly regulated may save the jobs and livelihoods of large numbers of poor people. If the livelihood benefits outweigh the calibrated risk, then the net positive benefit may increase. This approach can be useful in identifying alternative approaches and combinations of measures and weighing the potential benefits and externalities both positives and negatives to maximize net social benefits in achieving the broad social goals. Table 1 shows an example of qualitative assessment of different policy options to maximize complementary effects and minimize counter-productive effects in order to enhance the net societal benefits (Table 1).

**Table 1:** Example of a qualitative assessment of different policy options and actions.

**Proposed activities**	**Potential societal benefits**			**Externalities**		**Scale of effects (positive/negative)**		
	**Health**	**Economic**	**Environment**	**+/– ve effects**	**– ve effects**	**Short**	**Medium**	**Long**
Imposing lockdown	**+**	**–**	**+**	**+/–**	–	**+**	**–**	**+/–**
Increasing investment in health care	**+**	**+**	**+**	+/o	0	**+**	**+**	**+**
Increasing investment in education, skills development	**+**	**+**		+/o	0	**+**	**+**	**+**
Creating jobs in public construction of water infrastructure	**+**	**+**	**+**	+/o	0	**+**	**+**	**+**
Creating jobs in growing nutritious crops	**+**	**+**	**+**	+/o	0	**+**	**+**	**+**
Supporting jobs in growing tobacco for cash income	**–**	**+**	**–**	**–**	**–**	+	**–**	**–**
Withdrawing trade barriers	**+**	**+**	**+/–**	**+**		**+**	**+**	**+**
Subsidizing airline industries	**–**	**+**	**–**	**–**	**–**	**+**	**–**	**–**
Policies for withdrawing subsidies on fossil fuels	**+**	**+/–**	**+**	**+**		**+/–**	**+**	**+**

*+, positive effect; –, negative effect; o, no relationship; **+** ve, positive externalities; **–** ve, negative externalities*.

In additional to the fundamental criteria outlined above of potential societal benefits, externalities, and scale of positive and negative effects, three additional criteria should be taken into account in the selection of policies and investment decisions—coherence, compatibility, and congruence.

- **Coherence** is needed both among the different health related policy goals, and between these and any policies outside the health sector whose action may affect the outcome of the health sector goals. Ideally, the selected policies should contribute to achieving multiple policy goals
- **Compatibility** of policy options refers to the consistency in how they reinforce or undermine related policy goals and externalities and is needed to enable policies to contribute to achieving multiple health-related goals.
- **Congruence** refers to the ability of policy options and strategies to work together in a mutually supportive manner to help attain health sector and health-related non-health sector goals

### Aligning Policy Instruments to Improve Policy Coherence

Governments can use different policy instruments (the financial, regulatory, and market tools used to influence people's choices and behavior) and shape the incentive structure to achieve the desired social goals (10). Once the policy options to be implemented have been identified using a smart strategy approach, the best policy instruments need to be chosen and strategies and instruments aligned to maximize the potential for success in achieving the broad social goals in addition to the fundamental principles and criteria agreed (Figure 1).

#### Improving Policy Coherence

One way to align policy instruments is to improve policy coherence across health, economic, social, and environmental goals so that the policy instruments of one objective do not undermine those of another (15, 33). For example, supporting employment through growing tobacco for cash income in rural areas may increase income but can hinder the goal of achieving good health. Likewise, subsidizing chemical fertilizers and pesticides to increase crop productivity might result in water and air pollution and thus also defeat the goal of achieving good health (12). Similarly, encouraging plantation of erosive crops like cassava in a hill area to provide higher incomes in the short term, may exacerbate soil erosion and land degradation and undermine productivity and sustainability in the long-term.

#### Managing Externalities

Another way to improve policy coherence is to manage externalities. A positive (beneficial) or negative (harmful) externality is the consequence of an industrial or commercial activity which affects other parties without this being reflected in market prices (10, 37). For example, when bees kept for honey pollinate surrounding crops there is a positive externality for the owner of the crops. However, when industrial waste pollutes a water source and affects and pollutes fish stocks, downstream fisher communities experience a negative externality that reduces income and impacts health. Negative externalities resulting from production processes can include environmental pollution, overexploitation of natural resources, and degradation of ecosystems, all of which can affect the natural environment and planetary health, which in turn are closely related to human health (35, 38, 39).

Externalities arise because decisions on production or consumption of goods or services taken by a private investor or consumer are not designed to take into account the broader social consequences as these are not reflected in the market price (10). Conventional market mechanisms, and thus prices, are not designed to reflect the costs and benefits of social goods and services (for example costs of disposing of waste, of addressing environmental pollution, of treating ill health caused by a product) unless required by statutory instruments (such as a surcharge or tax related to disposal costs or health impact). Designing policy instruments to maximize positive and minimize negative externalities can be instrumental for achieving broad social goals (12). Policy instruments need to be chosen that create disincentives for negative externalities through taxes, fines, or fees, and encourage positive externalities through subsidies, rewards or other incentives.

#### Reconciling Private and Social Interests

Another result of private decision makers not being required to consider the broader social consequences of their decisions is that social goods tend to be under produced and private goods over produced (19, 37). The divergence of private and social interests often leads to sub-optimal production of social goods such as public health, public transport, clean air, clean water, education, research, and innovation, which generate positive externalities for society. Private investors cannot directly capture the full benefits generated through social goods and thus investment in social goods lies outside their interests. However, it is difficult for governments to deliver the required social goods alone, and private sector participation is important for filling the gap between public need and financial capacity. Appropriate incentive mechanisms can be developed to encourage private investment in social goods, including improvements in the healthcare system (37, 40). Choices that internalize such external costs should be considered carefully and prioritized. When incentives are not enough, policy reforms that regulate the unsustainable use of resources and impose standards and procedures to internalize external costs and control pollution should be prioritized. For example, regulating trade in wild animals and direct contact with animal parts reduces the exposure of humans to contact with viruses and other pathogens hosted by those species. Similarly, raising the cost of fossil fuels can help in reducing air pollution and improving air quality, while providing subsidies for private intensive care units can reduce investment requirements, and thus support public health provision (12). Different types of policy instruments that reconcile private and social interests, from incentive-based mechanisms to regulation, should be prioritized.

Thus, it is critically important to select the policy instruments strategically and arrange them carefully so that they work together and are mutually supportive in reaching health-related policy goals. Considerations on policy tools can involve purposeful arrangements of policy instruments in such a way as to generate positive interactions between them. However, in choosing policy instruments, the cultural and operational capacity needs to be considered carefully as different instruments require different levels of operational capacity to implement and not all instruments are feasible in every socio-cultural context (13).

### Integrating Long-Term Sustainability in Policy Decisions

No matter what challenges need to be addressed in the short-term, government policies and actions should take into account the need for sustainability in the long-term. Thus, policy choices should focus both on resolving urgent needs and on ensuring long-term resilience and sustainability while taking to account the fundamental principles and criteria agreed as the fundamental basis (Figure 1).

#### Factoring Sustainability Into Short-Term Policy Choices

The COVID-19 pandemic provides an opportunity to take a broad look at factoring sustainability—economic, social, and environmental—into policy choices in order to create more resilient societies (35, 38). This requires strategic thinking and a systematic assessment of policy options and strategies for long-term investment to ensure that the short-term actions result in long-term benefits. Some of the short-term support can be linked to long-term economic growth by appropriate conditions that improve the social and environmental conditions for health (12, 41). For example, food for work programs can be attached to programs for adapting or constructing local infrastructure to maintain social distancing, thus helping poor households to cope with vulnerability while building assets that are essential for society. Similarly, requirements to include energy efficiency in building designs can be linked to support provided to building construction companies to restore jobs, thus providing job restoration in the short-term and climate benefits in the long-term.

#### Improving Long-Term Productivity and Resilience

The short-term focus will be on addressing the impacts of the pandemic and, following the direct health-related activities, is likely to focus on employment generation and restoration of jobs. However, long-term investment decisions should also be considered. Investing in a balanced portfolio of physical, human, social and natural capital will help improve long-term productivity and resilience, and thus build capacity to deal with future challenges and mitigate the impact of future pandemics and disasters (12, 41, 42). For example, investment in health, education, skills development, innovation, technological upgrading, and green infrastructure and natural capital will increase productive capacity and provide sustainable returns for future generations (11, 19). Investment in social protection and job creation will be needed to protect the vulnerable in the short term, but policy priorities could gradually shift to reducing the environmental risks affecting human health and vulnerability to climate change. Protecting and enhancing natural capital such as forests, soils, water resources, ecosystems, biodiversity, air quality, and climate can support human health and productivity and improve long-term resilience (41, 43). For example, investment in green infrastructure such as renewable energy can supply clean energy and improve air quality, which leads to long-term health benefits and positive climate outcomes (39).

In striving for sustainability, policy choices, and investment decisions should be arranged strategically in such a way that they not only address immediate problems but also build long-term resilience.

## Conclusion

Identifying the best set of policies and instruments to address COVID-19 challenges and aligning them with broader social goals will be critically important for sustainable recovery from the pandemic and resilient society. The way in which governments set their priorities, prioritize policies and programs, and coordinate activities will affect the outcome. Poorly identified policy choices are likely to be ineffective in addressing the health, economic, social, and environmental challenges and harnessing the potential long-term economic and environmental benefits. This paper presents a framework for identifying and prioritizing policy actions to address the COVID-19 challenges and ensure sustainable recovery. The framework outlines principles and criteria, and a suggested approach, for assessing and prioritizing policy choices in planning and decision making. It offers guidelines for developing shared policy goals, identifying smart strategies, aligning policy instruments, and factoring sustainability into short and long-term policy decisions.

In contrast to the common practice of evaluating policy outcomes after implementation, this framework enables policy makers to think ahead and assess the anticipated consequences of different policy options and their positive and negative cross-sectoral implications, which is critically important for developing a coherent and integrated set of policy decisions in the uncertain volatile situation of the COVID-19 pandemic. The framework can help governments to prioritize policy choices and allocate limited resources in such a way that they are directed toward actions that generate synergy and co-benefits, have multiplier effects, and achieve interconnected solutions for health, the economy, and the environment.

Enhancing cross-sectoral integration and improving policy coherence is a challenging task requiring strong commitment from governments. A major prerequisite for using the framework is to establish a multi-sectoral coordination body with the capacity to mobilize and build partnership, consensus, and ownership among the multiple government and non-government agencies and thus increase horizontal and vertical policy coherence and strengthen policy coordination for collective action. The suggested framework is generic, and could be further developed using quantitative tools for detailed analysis and quantification of the complementarities and trade-offs presented in Table 1. Although this framework is intended to address COVID-19 challenges, this can be customized and used in different policy arenas in managing cross-sectoral and interconnected challenges. Cross-sectoral collaboration and problem solving is demanding knowledge and capacity in managing inter-sectoral dynamics. In designing the detailed policies and strategies, cultural values, and operational capacity—including leadership, coordination, and implementation—and political realities will need to be considered (13).

## Data Availability Statement

The original contributions presented in the study are included in the article/supplementary material, further inquiries can be directed to the corresponding author/s.

## Author Contributions

The author confirms being the sole contributor of this work and has approved it for publication.

## Conflict of Interest

The author declares that the research was conducted in the absence of any commercial or financial relationships that could be construed as a potential conflict of interest.
